# Draft genome sequences of *Staphylococcus aureus* clinical isolate (SA2-IRQ) from a patient with an ear infection

**DOI:** 10.1128/mra.00845-26

**Published:** 2026-09-02

**Authors:** Ibrahim A. Ahmed, Zaid A. Thabit, Yaseen I. Mamoori, Nisreen S. Hasan

**Affiliations:** 1 Applied Biology Department, College of Biotechnology, Al-Nahrain University 54645, Baghdad, Iraq; 2 Biotechnology Research Center, Al-Nahrain University 54645, Baghdad, Iraq; 3 Department of Molecular and Medical Biotechnology, College of Biotechnology, Al-Nahrain University 54645, Baghdad, Iraq; 4 Department of Anesthesia Techniques, College of Health and Medical Techniques, Al-Esraa University 554706, Baghdad, Iraq; Fluxus Inc., Sunnyvale, California, USA

**Keywords:** virulence factors, ear infection, genome sequence, SA2-IRQ

## Abstract

In the current study, we present the genome sequence of *Staphylococcus aureus* isolated from a patient suffering from an ear infection. The bacterial genome was assembled into 26 contigs, comprising a total of 2,774,206 bp with a GC content of 32.5% and encoded approximately 2,782 genes, including multiple virulence-associated genes.

## ANNOUNCEMENT

Ear infections, also known as otitis, are widespread infections that affect many people worldwide (1, 2). Several studies have reported that *Staphylococcus aureus* is the most common pathogenic bacterium associated with ear infection*s* (3, 4). Whole-genome sequencing (WGS) enables comprehensive characterization of antimicrobial resistance, virulence, and epidemiological features, supporting clinical diagnosis and infection control (5, 6).

*Staphylococcus aureus* (SA2-IRQ) was isolated from an ear swab collected from a 19-year-old patient with an ear infection at Al-Kadhimiya Hospital in Baghdad. The ear swab was inoculated on mannitol salt agar (MSA) plates, followed by incubation at 37°C overnight. After that, a single colony was selected and cultivated overnight in brain heart infusion (BHI) broth at 37°C with agitation at 150 rpm. The identity of the bacterium was determined by the VITEK 2 system and confirmed by amplification of the *nuc* gene (7). Prior to genomic DNA extraction, the bacterium was cultured overnight in BHI broth at 37°C with agitation at 150 rpm. Bacterial genomic DNA was isolated using the Geneaid DNA Isolation Kit for Bacteria (Geneaid, Taiwan). The extracted genomic DNA was analyzed for concentration and purity using a NanoDrop 2000 UV–Vis spectrophotometer (Thermo Fisher Scientific, USA), and its integrity was checked by agarose gel electrophoresis. Afterward, the sample was submitted to Apical Scientific for genome sequencing (Selangor, Malaysia).

The VAHTS Universal Plus DNA Library Prep Kit (No. ND801-C5, Vazyme Biotech Co., Ltd., China) was used to create the sequence libraries on the Illumina NovaSeq X Plus platform (2 × 150 bp, chemistry V1.5), resulting in 6,946,940 paired-end reads. FastQC (v0.12.1) (8) was used to assess the data quality, and Fastp (v0.24.0) (9) was used for quality filtering. After quality filtering, the sequencing reads underwent assembly using Unicycler v0.5.1 (10), which incorporates with the SPAdes algorithm (11). The quality of the genome assembly and completeness were evaluated using CheckM2 (v1.1.0) (12) and Quast (v5.3.0) (13). Multilocus sequence typing was carried out using MLST v2.22.0 (14).

**Fig 1:**
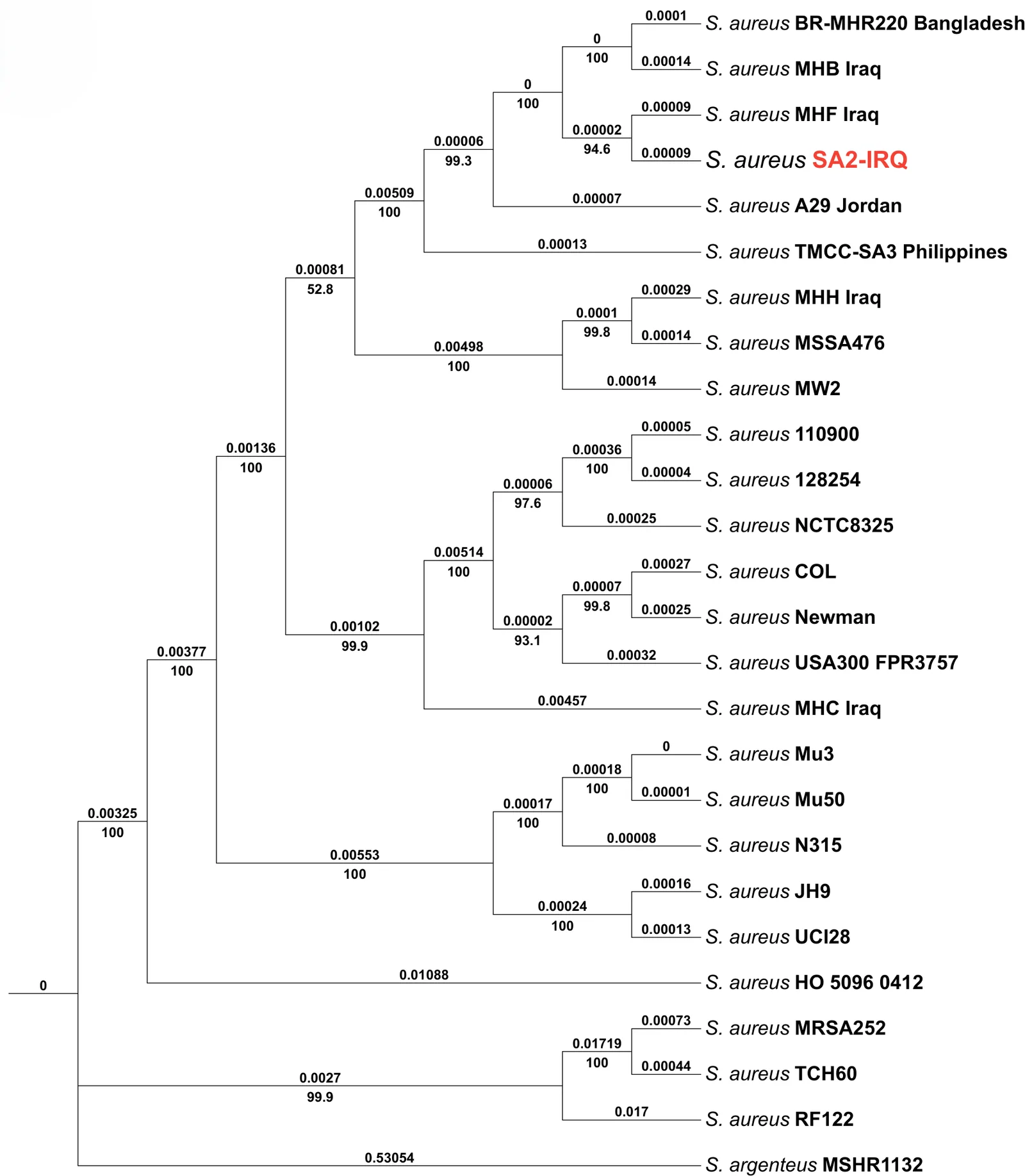
Maximum-likelihood phylogenetic analysis of SA2-IRQ. The maximum-likelihood phylogenetic tree inferred from recombination-filtered core genome SNPs.

NCBI Prokaryotic Genome Annotation Pipeline (v6.10) (15) was used to functionally annotate the genome, predicting 2,782 total genes as described in Table 1. Antimicrobial resistance genes were predicted by AMRFinderPlus (16), and virulence-associated genes were identified using the VFDB database (17). A recombination-corrected core genome single-nucleotide polymorphism (SNP) alignment was generated using Snippy (18) and Gubbins (19) and subsequently employed for maximum-likelihood phylogenetic reconstruction in IQ-TREE 2 (20) under the best-fit model selected by ModelFinder (21) with ascertainment-bias correction. The reliability of the phylogenetic tree was validated using 1,000 ultrafast bootstrap replicates (22). The tree was rooted using *Staphylococcus argenteus* MSHR1132 as an outgroup and visualized in iTOL (23) (Fig. 1). Phylogenomic analysis placed the isolate within the ST6 (Table 1).

**TABLE 1:** Genomic characteristics of *Staphylococcus aureus* SA2-IRQ^*a*^

Features	Values
Genome size (bp)	2,774,206
GC content (%)	32.73
No. of contigs	26
L50 (no. of contigs)	3
N50 (bp)	497,499
Genome coverage (×)	717.69
Genes (total)	2,782
CDSs (total)	2,717
Gene (coding)	2,644
Genes (RNA)	65
rRNAs	2, 1, 2 (5S, 16S, 23S)
tRNAs	56
ncRNAs	4
Pseudogenes (total)	73
Completeness (%)	100.0
Sequence typing	ST6
Resistance genes	*mepA*, *tet (38)*, *mecA*, *blaI*, *blaZ*
Virulence factors	*hly/hla, isdG, srtB, isdF, isdE, isdD, isdC, isdA, isdB, sspA, sspB, sspC, vWbp, clfA, sdrE, sdrD, sdrC, hld, hlb, lukF-PV, harA, ebp, set25, set24, set23, set22, set20, set19, set18, set17, set16, geh, esaG, esaG, esaG, esaG, esaD, esxD, esaE, esxB, esxC, essC, essB, esaB, essA, esaA, esxA, isdI, cap8P, cap8O, capN, cap8M, cap8L, cap8K, cap8J, cap8I, cap8H, cap8G, cap8F, cap8E, cap8D, cap8C, cap8B, capA, spa, adsA, eap/map, scn, sak, sea, hlgB, hlgC, hlgA, sbi, icaR, aur, clfB, fnbA, hysA, lip, icaC, icaB, icaD, icaA, fnbB*

^*a*^ CDSs, coding sequences; ncRNA, noncoding RNA.

## Data Availability

The SA2-IRQ genome sequence was submitted to GenBank under accession no. JBZGVZ000000000, BioProject PRJNA1477650, BioSample SAMN60763899, Assembly GCF_058374755.1, and raw reads accession no. SRR39122646.
